# Factors affecting adherence to antiretroviral therapy among pregnant women in the Eastern Cape, South Africa

**DOI:** 10.1186/s12879-018-3087-8

**Published:** 2018-04-13

**Authors:** Oladele Vincent Adeniyi, Anthony Idowu Ajayi, Daniel Ter Goon, Eyitayo Omolara Owolabi, Alfred Eboh, John Lambert

**Affiliations:** 1 Department of Family Medicine & Rural Health, Faculty of Health Science, Walter Sisulu University, Mthatha/East London Hospital Complex, Cecilia Makiwane Hospital, East London, South Africa; 2 Department of Sociology, Faculty of Social Sciences & Humanities, University of Fort Hare, 50, Church Street, East London, South Africa; 3 Department of Nursing Sciences, Faculty of Health Sciences, University of Fort Hare, East London, South Africa; 4 Department of Nursing Sciences, Faculty of Health Sciences, University of Fort Hare, East London, South Africa; 5 Department of Sociology, Kogi State University, Anyigba, Kogi, State P.M.B 1008 Nigeria; 6 Department of Infectious Diseases, Medicine and Sexual Health, Mater, Rotunda and University College, Dublin, Ireland

**Keywords:** Adherence, Non-adherence, HIV, Antiretroviral therapy, Elimination of mother-to-child transmission, Prevention of mother-to-child transmission, Stigma, South Africa

## Abstract

**Background:**

Context-specific factors influence adherence to antiretroviral therapy (ART) among pregnant women living with HIV. Gaps exist in the understanding of the reasons for the variable outcomes of the prevention of mother-to-child transmission (PMTCT) programme at the health facility level in South Africa. This study examined adherence levels and reasons for non-adherence during pregnancy in a cohort of parturient women enrolled in the PMTCT programme in the Eastern Cape, South Africa.

**Methods:**

This was a mixed-methods study involving 1709 parturient women in the Eastern Cape, South Africa. We conducted a multi-centre retrospective analysis of the mother-infant pair in the PMTCT electronic database in 2016. Semi-structured interviews of purposively selected parturient women with self-reported poor adherence (*n* = 177) were conducted to gain understanding of the main barriers to adherence. Binary logistic regression was used to determine the independent predictors of ART non-adherence.

**Results:**

A high proportion (69.0%) of women reported perfect adherence. In the logistic regression analysis, after adjusting for confounding factors, marital status, cigarette smoking, alcohol use and non-disclosure to a family member were the independent predictors of non-adherence. Analysis of the qualitative data revealed that drug-related side-effects, being away from home, forgetfulness, non-disclosure, stigma and work-related demand were among the main reasons for non-adherence to ART.

**Conclusions:**

Non-adherence to the antiretroviral therapy among pregnant women in this setting is associated with lifestyle behaviours, HIV-related stigma and ART side-effects. In order to eliminate mother-to-child transmission of HIV, clinicians need to screen for these factors at every antenatal clinic visit.

## Background

The introduction of antiretroviral medications has significantly reduced deaths attributable to AIDS as well as vertical and horizontal transmissions [1–4], especially in South Africa [5]. Despite this progress, non-adherence to antiretroviral therapy (ART) remains a barrier to achieving its maximum benefits, especially that of elimination of mother to child transmission of HIV [6, 7]. High-levels of sustained adherence have been demonstrated to be directly associated with a decline in the babies’ HIV acquisition risk [8], and viral load suppression [9] and increased life expectancy in the mother [2]. In contrast, poor adherence to ART has been associated with poor treatment outcomes, emergence of resistance, patients’ dissatisfaction, increased healthcare expenditure, and avoidable deaths [6, 9–12].

Despite the evidence showing pregnant women having the highest level of adherence to ART [13], a low level of adherence has been reported in some settings among pregnant women [14]. The adherence rate among pregnant women varies across different settings (within and across countries) from 35% to 93.5% [15]. A study reported low adherence levels among pregnant women in rural Kwazulu Natal, South Africa [14]; however, a high level of adherence (90%) was reported in Kenya [16]. An adherence level of 73% during pregnancy was reported in Malawi, but dropped to 66% by three month postpartum [17]. Adherence levels of 75% were reported among US women during pregnancy, but reduced to 65% at six weeks postpartum [18]. There is little information on adherence among pregnant women on ART in the Eastern Cape, South Africa. This epidemiological data will guide the on-going reforms by the Department of Health within the Eastern Cape towards meeting the national and global target of achieving elimination of mother-to-child HIV transmission.

Previously reported barriers to antiretroviral (ARV) medication adherence include: ART side-effects, social stigma, depression, non-disclosure of HIV status, unemployment, food insecurity, alcohol/substance abuse, alternative forms of therapy, inadequate follow-ups, stock outs, work and family responsibilities, low self-efficacy, low treatment satisfaction and distance to clinics [19–24]. The factors associated with non-adherence also vary contextually. For instance, knowledge was linked to non-adherence in Israel [25], while younger age, rural residence and substance use were important in Kenya [16]. In Malawi, lack of emotional and financial support from husband, inadequate counseling and internal migration were the reported reasons for non-adherence to ART [26].

A study conducted in Romania found that ART side effects, low self-efficacy, low treatment efficacy, low treatment satisfaction and emotional distress are the main barriers to ART adherence [24]. In contrast, the review of reasons for non-adherence to ART in sub-Saharan Africa indicate that giving birth at home, quality and timing of HIV testing and counseling, fear of stigma, lack of male involvement, non sero-status disclosure, young age and lack of education are linked to low adherence [15]. A study conducted on caregivers’ perspectives on non-adherence shows that insufficient patient education and social support, patient dissatisfaction with healthcare services, socioeconomic factors, and tension between ART and alternative medicine are the main reasons for non-adherence [23].

The Eastern Cape Provincial Department of Health is committed to improving the maternal and infant indicators in line with the national target, and high adherence level is needed to achieve these targets. Many studies on adherence were quantitative [14, 16, 17, 22] and did not allow for in-depth understanding of the reasons for ART non-adherence. Using a mixed methods design, this study examined the level of adherence to ART during pregnancy, and explored correlates of non-adherence and reasons for non-adherence in the Eastern Cape Province, South Africa. Concurrent triangulation of quantitative and qualitative methods was used to overcome the weakness of a single design. The findings from qualitative study were used to explain, validate, confirm and corroborate the findings of the quantitative study.

## Methods

### Study design and settings

We conducted a sub-analysis of the electronic database of a larger study (East London Prospective Cohort Study) [27]. This multi-centre study was conducted in three large maternity facilities in the Buffalo/Amathole districts of the Eastern Cape Province, South Africa. These facilities provide healthcare services for a combined population of 1,674,637 people [28] in the two districts with rural, semi-urban and urban demographics resembling the entire Eastern Cape population. This study adopted a mixed-methods descriptive design involving the use of a questionnaire and semi-structured interviews for eliciting information from research participants. The mixed-methods design involved a concurrent triangulation of both quantitative and qualitative data. The rationale for triangulation of qualitative and quantitative design was to use the findings of the qualitative study to explain the findings of the quantitative study. A total of 1709 participants were recruited and interviewed using a pre-validated structured questionnaire. The questionnaire was piloted among 20 women who were not included in the study. For the qualitative study, a total of 177 purposively selected HIV infected parturient women, who self-reported non-adherence to ARV, were interviewed. Each interview lasted for an average of twenty minutes. All interviews were audio-taped and later transcribed.

#### Participants

All HIV infected parturient women who delivered their index pregnancy at the maternity centres in Frere, Cecilia Makiwane and Bisho hospitals between September 2015 and May 2016 were recruited into the electronic database. Peri-partum women who are HIV negative were excluded from the study.

#### Measures

##### ART adherence

Adherence to ART was measured using two items, which probed on-time monthly pharmacy refill of medications (Yes/No) and self-report of adherence during index pregnancy (Yes/No). Adherence measure by drug-refill has been demonstrated to be a useful early warning indicator of virological and immunological failure among HIV-infected individuals [29]. Both items were summed to provide an ordinal measure of adherence, which ranged from 0 to 2, with a score of 0 signifying good adherence while 1 to 2 signified non-adherence. We used Cronbach’s alpha reliability test to examine the level of reliability of our adherence measure and the result yielded a moderate level of reliability (Alpa:0.61). Our measure of adherence yielded good internal consistency.

##### Correlates of ARV adherence

Participants reported their age, level of education, marital status, employment status, and place of residence. Participants also indicated their lifestyle behaviours; cigarette smoking and alcohol consumption status before and during the index pregnancy. In addition, participants were asked to report the number of deliveries (parity), gestational age at booking, HIV serostatus at booking, and whether they were already on ART at booking. Also, participants were asked whether they had disclosed their HIV serostatus to their sexual partner or any family members. Data on trimester at booking were retrieved from participants’ clinical records. Incomplete data of some variables due to non-response or lack of information in the medical records were noted and were subsequently, excluded from the final analysis.

##### Reasons for non-adherence

To explore the reasons for non-adherence in the qualitative study, participants were asked to state their reason(s) for non-adherence to their ARV medications and their responses were recorded.

### Statistical analysis

The data generated were captured in the electronic database and were imported into Statistical Packages for Social Sciences (SPSS version 24 for Windows, Chicago, IL, USA). Simple frequencies of all variables were computed. A chi-square test was applied to examine association between demographic, behavioural characteristics, and non-adherence. Binary logistic regression was used to determine the independent predictors of ART non-adherence. The level of significance was set at alpha = 0.05. Only variables with a *p*-value less than or equals to 0.10 were included in the multivariate analysis. Disclosure to sexual partner correlated with disclosure to a family member, thus, was not included in the multivariate analysis. The model was adjusted for employment status, HIV status at booking, on ART at booking and disclosure to sexual partner.

### Qualitative data analysis

The qualitative data were analysed using recursive abstraction [30]. The transcribed qualitative data were read, coded and grouped according to emerging themes. Sample of the transcription was verified for accuracy by two authors. The recursive abstraction analysis involved six steps. In the first step, every phrase and sentence of interest was highlighted. We grouped the data by question to make sure each individual point is separate. This was done by transferring the highlighted data into a table, with the question topic on the rows and interviewee’s response in the columns. Data were read reflexively before coding and grouping of codes into themes. The codes and emerging themes were shown to two other authors for validation.

#### Ethical clearance

Walter Sisulu University Ethics Committee granted approval for this study. Also, the Eastern Cape Department of Health gave permission for the implementation of the study. The heads of the respective health facilities gave permission prior to data collection. Participation was voluntary. All participants provided written informed consent to indicate their voluntary participation in the study before administering any instrument. We obtained parental consent for three participants below 16 years, who assented to participate in the study. The researchers ensured that participants understood their rights of voluntary participation, anonymity and confidentiality. The researcher ensured the rights of participants during and after the study.

## Results

Most of the study participants were single (69.5%), had completed grade 12 schooling (86.5%), were unemployed (74.5%), and were non-smokers (89.5%) (See Table 1). The majority of the participants booked during the second trimester (73.2%), knew their HIV positive status before booking (80.9%) and were already on ART (58.4%).

**Table 1:** Demographic characteristics of study participants

Variables	Frequency (*n* = 1709)	Percentage
Marital Status		
Married	312	18.3
Single	1187	69.5
Co-habiting	186	10.9
Divorce/Separated	24	1.4
Place of Residence		
Rural	585	34.2
Semi-urban	792	46.3
Urban	332	19.4
Educational Level		
No formal education	5	0.3
Grade 1–6	115	6.7
Grade 7–12	1479	86.5
Tertiary	110	6.4
Employment status		
Unemployed	1277	74.7
Employed	432	25.3
Smoking status		
Smoked during pregnancy	100	5.9
Quit smoking during pregnancy	80	4.7
Never smoked	1529	89.5
Alcohol use		
Drank during pregnancy	235	13.8
Quit drinking during pregnancy	431	25.2
Never drank alcohol	1043	61.0
^a^Trimester at booking		
First	110	6.8
Second	1177	73.2
Third	322	20.0
Status at first visit		
Positive	1382	80.9
Negative	94	5.5
Unknown	233	13.6
On ART at booking	998	58.4

*ART* antiretroviral therapy, ^a^Incomplete data on trimester at booking (*n* = 100)

### Proportion of women who were adherent

The majority of the participants (69.0%) reported adherence to ART (Table 2). Adherence rates were highest among pregnant women who were married (76.9%), had a minimum of grade six level of education (82.5%), were non-smokers (71.1%), non-alcoholic (76.1%), who booked during the second trimester (71.6%), had disclosed their HIV status to their sexual partner (70.3%) or a family member (70.9%), and reported positive HIV status at booking (70.9%). In contrast, the adherence rate was lowest among women who smoked during pregnancy (39.3%).

**Table 2:** Demographic and behavioural correlates of ART adherence*

Variables	Adherence (*n* = 1098; 69%)	Non-adherence (*n* = 494; 31.0%)	*p*-value
Age			
< 25 years	230(63.5)	132(36.5)	0.008
≥ 25 years	863(70.4)	362(29.6)	
Marital Status			
Married	230(76.9)	69(23.1)	< 0.001
Single	760(69.5)	333(30.5)	
Co-habiting	95(54.0)	81(46.0)	
Divorce/Separated	13(54.2)	11(45.8)	
Educational Level			
No formal education	1(20.0)	4(80.0)	0.002
Grade 1–6	85(82.5)	18(17.5)	
Grade 7–12	947(68.4)	438(31.6)	
Tertiary	65(65.7)	34(34.3)	
Employment Status			
Unemployed	815(68.5)	374(31.5)	0.286
Employed	283(70.2)	120(29.8)	
Smoking Habit			
Smoked during pregnancy	33(39.3)	51(60.7)	< 0.001
Quit smoking during pregnancy	48(61.5)	30(38.5)	
Never smoked	1017(71.1)	413(28.9)	
Alcohol Use			
Drank during pregnancy	92(44.0)	117(56.0)	< 0.001
Stopped during pregnancy	266(64.9)	144(35.1)	
Never drank	740(76.1)	233(23.9)	
HIV status at booking			
Positive	914(70.9)	376(29.1)	0.003
Negative	53(63.9)	30(36.1)	
Unknown	131(59.8)	88(40.2)	
On ART at booking			
No	263(71.7)	104(28.3)	0.373
Yes	659(70.6)	275(29.4)	
Disclosure to partner			
No	256(64.8)	139(35.2)	0.024
Yes	836(70.3)	353(29.7)	
Disclosure to a family member			
No	188(60.8)	121(39.2)	< 0.001
Yes	906(70.9)	372(29.1)	
Trimester at booking			
First	95(57.2)	71(42.8)	< 0.001
Second	843(71.6)	335(28.4)	
Third	160(64.5)	88(35.5)	

*ART* antiretroviral therapy, *Incomplete data were excluded from the univariate analysis

In the binary logistic regression, after adjusting for confounding factors (disclosure to sexual partner, employment status, HIV status at booking and on ART at booking), only marital status, grade 1–6 education level, cigarette smoking, alcohol use, and non-disclosure to a family member were independent predictors of non-adherence (See Table 3). Relative to non-smokers, women who smoked during pregnancy (OR: 1.91; CI:1.10–3.33) had increased odds of reporting non-adherence. Likewise, women who had not disclosed their status to a family member had increased odds (OR:1.50; CI:1.13–1.98) of non-adherence compared to women who had disclosed their HIV status to a family member. Women who drank alcohol during pregnancy were 3.19 (CI:2.23–4.56) times more likely to report non-adherence to ART compared to women who never drank alcohol. Compared to married women, co-habiting (AOR: 1.71; CI: 1.11–2.64) and divorce/separated women (AOR: 3.24; CI: 1.33–7.91) had higher odds for non-adherence to ART. Relative to women who had tertiary level of education, women who had grade 1–6 level of education (AOR:0. 51; CI: 0.26–0.98) had lesser odds of reporting non-adherence to ART.

**Table 3:** Adjusted and unadjusted binary logistic regression analysis showing determinants of non adherence to ART

Variables	Adherence (*n* = 1098; 69%)	Non-adherence (*n* = 494; 31.0%)	Unadjusted odd ratio	Adjusted odd ratio
Age				
< 25 years	230(63.5)	132(36.5)	1.36(1.06–1.74)*	0.80(0.61–1.04)
≥ 25 years	863(70.4)	362(29.6)	Ref	Ref
Marital Status				
Married	230(76.9)	69(23.1)	Ref	Ref
Single	760(69.5)	333(30.5)	1.40(1.04–1.87)*	1.22(0.89–1.66)
Co-habiting	95(54.0)	81(46.0)	2.38(1.59–3.56)***	1.71(1.11–2.64)*
Divorce/Separated	13(54.2)	11(45.8)	2.79 (1.16–6.70)*	3.24(1.33–7.91)*
Educational Level				
No formal education	1(20.0)	4(80.0)	6.97(0.75–64-86)	4.79(0.47–48.91)
Grade 1–6	85(82.5)	18(17.5)	0.48(0.25–0.90)*	0.51(0.26–0.98)*
Grade 7–12	947(68.4)	438(31.6)	0.91(0.59–1.39)	0.79(0.50–1.24)
Tertiary	65(65.7)	34(34.3)	Ref	Ref
Employment Status				
Unemployed	815(68.5)	374(31.5)	0.94(0.73–1.20)	
Employed	283(70.2)	120(29.8)	Ref	
Smoking Habit				
Smoked during pregnancy	33(39.3)	51(60.7)	4.0(2.45–6.53)***	1.91(1.10–3.33)*
Quit smoking during pregnancy	48(61.5)	30(38.5)	1.51(0.94–2.44)	1.12(0.66–1.88)
Never smoked	1017(71.1)	413(28.9)	Ref	Ref
Alcohol Use				
Drank during pregnancy	92(44.0)	117(56.0)	3.80(2.76–5.24)***	3.19(2.23–4.56)***
Stopped duringpregnancy	266(64.9)	144(35.1)	1.55(1.20–1.99)**	1.38(1.05–1.80)*
Never drank	740(76.1)	233(23.9)	Ref	Ref
HIV status at booking				
Positive	914(70.9)	376(29.1)	Ref	
Negative	53(63.9)	30(36.1)	1.08(0.66–1.77)	
Unknown	131(59.8)	88(40.2)	1.39 (1.02–1.89)*	
On ART at booking				
No	263(71.7)	104(28.3)	1.28(0.98–1.67)	
Yes	659(70.6)	275(29.4)	Ref	
Disclosure to partner				
No	256(64.8)	139(35.2)	1.52(1.17–1.97)**	
Yes	836(70.3)	353(29.7)	Ref	
Disclosure to a family member				
No	188(60.8)	121(39.2)	1.30(1.02–1.66)*	1.50(1.13–1.98)*
Yes	906(70.9)	372(29.1)	Ref	Ref
Trimester at booking				
First	95(57.2)	71(42.8)	1.22(0.69–2.16)	1.34(0.74–2.42)
Second	843(71.6)	335(28.4)	0.73(0.55–0.97)*	0.79(0.58–1.06)
Third	160(64.5)	88(35.5)	Ref	Ref

Ref: Reference; Model adjusted for disclosure to sexual partner, HIV status at booking, on ART at booking and employment status, *** represents *p*-value <0.000, ** represents *p*-value less than 0.01, * represent *p*-value < 0.05

### Reasons for non-adherence to ART

**Fig. 1:**
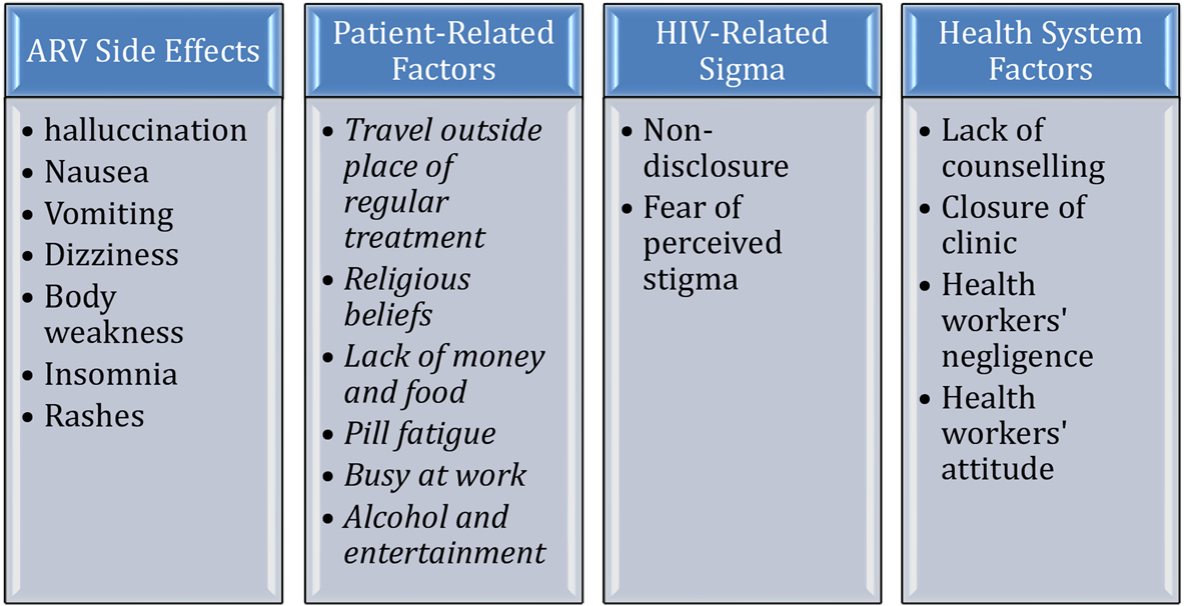
Reasons for Non-adherence among pregnant women on ART

The demographic characteristics of participants of the qualitative study are presented in Table 4. The main findings on the reasons for non-adherence in the analysis of the qualitative data are presented under four main themes: ART side effects, patient-related, HIV-associated stigma and health system factors (Fig. 1).

**Table 4:** Demographic characteristics of participants in the qualitative study

Variables	Frequency	Percent
Age		
Below 21	15	8.5
21–30	86	48.6
Above 30	76	42.9
Marital Status		
Married	25	14.1
Single	122	68.9
Co-habiting	27	15.3
Divorce/Separated	3	1.5
Place of Residence		
Rural	57	32.2
Semi-urban	90	50.8
Urban	30	17.0
Educational Level		
No formal education	2	1.1
Grade 1–6	8	4.5
Grade 7–12	160	90.3
Tertiary	7	4.0
Employment status		
Unemployed	148	83.6
Employed	29	16.4
Trimester at booking		
First	13	7.3
Second	130	73.4
Third	34	19.2
Status at first visit		
Positive	154	87.0
Negative	3	1.7
Unknown	20	11.3

#### Theme 1: ART side effects

Many women stated that they stopped their medications due to ARV side effects. The commonly reported side effects included hallucinations, nausea, vomiting, dizziness, body weakness, insomnia and rashes. Vomiting was the most commonly reported ART side effect. The view of a 25-year-old woman, who was initiated on ART during the index pregnancy, shed some light on the plight of women that experienced hallucinations:

*“After taking the medication, I would feel dizzy. I see weird things like snakes or hear voices calling my name at night. I was constantly having nightmares, so I decided to stop taking the treatment.”*

Some other women, who reported hallucinations, also stated that the medications made them feel dizzy, they experienced psychosis, had bad dreams, and saw dead people.

#### Theme 2: Patient-related factors

The reasons for ART non-adherence were mostly patient-related factors. These factors are explained under the sub-themes below:

##### Travel outside place of regular treatment

The second most reported reason for non-adherence among pregnant women was travel outside the place of regular treatment. Many women experienced difficulties in refilling their medications when they travelled outside/away from their current facility where they received treatment. Often, they travelled without their clinic book or transfer letters, which are a pre-requisite for receiving treatment in health facilities in South Africa. A woman who was turned down after requesting medications at a facility closest to her during her travels recounted:

*“I went to Transkei for 3 months, and I forgot to take my treatment book along; so the nurse in Transkei refused to give treatment without seeing my treatment book.”*

Women who visited Gauteng, Cape Town, Port Elizabeth and Mpumalanga also reported experiencing denial of treatment due to unavailability of their treatment books. Women who relocated to a new community or travelled to their rural homes without getting a transfer letter also stated that they defaulted because nurses in their new communities would not give them treatment without seeing their transfer letters. This view was captured by the response of a 23-year-old woman who was diagnosed with HIV during her index pregnancy:

*“I moved to a new community and I could not afford taxi fare to come back for my transfer letter.”*

Likewise, some women appeared to have purposefully left their medications behind when they visited their rural homes.

##### Religious beliefs

Some women stated that they stopped taking their medications because their pastors had cured them. Likewise, some women were given traditional medicine and were told not to use ART concurrently with the traditional medicine.

##### Lack of money and food

Another patient’s related factor, which is preventing women from adhering to their medication, was lack of money and food. A few women complained that the clinic was too far and they lacked the money for transportation to regularly pick up their medications. The view was expressed in the response of a woman who had previously been diagnosed, prior to her index pregnancy:

*“I stopped taking my treatment because I have no food and no money to go to the clinic.”*

##### Pill fatigue

Other women reported that they were simply tired of taking medications.

##### Busy at work

Another patient-related factor reported was interference of work with clinic schedules. Some of the women interviewed mentioned that drug pick-up schedules interfered with their work. Few women stated that they were busy at work and could not go to the clinic to fetch their medications. This view was captured by the response of a 33-year-old woman who stated:

*“I stopped using my medication because I got a domestic job where I could not leave work. I was a sleep-in helper.”*

##### Alcohol and entertainment

Some women reported that abuse of alcohol often made them forget to use their medications. This often happened especially over weekends when people club, attend social gatherings and drank a lot. Also, a few women stated that they did not adhere to ART during the festive period when they drank and attended social gatherings regularly. The response of a 33-year-old woman captured this view:

*“I defaulted for 3 months because it was the festive season and I was drinking and partying without knowing that I was already pregnant.”*

#### Theme 3: HIV-related stigma

The fear of enacted HIV stigma still remained and often prevented women from adhering to their medications. Some of the women interviewed stated that non-disclosure and fear of perceived stigma prevented them from using their medications. Few women who have not disclosed their status to their parents and sexual partners reported that they often defaulted on their medications because they had not disclosed their status; it was difficult for them to use their medication without being caught in the act. A 25-year-old woman stated:

“*I did not want to get caught taking the pills because of stigma.”*

Another woman stated:

*“I was scared to tell my mother about my status and she was always there when it was time to take the medications.”*

#### Theme 4: Health system factors

The analysis of the responses of the participants suggested that reasons for non-adherence to ARV medication were not limited to patient-related factors or HIV-related stigma. Health facility-related factors such as lack of counselling on adherence, closure of clinics, health workers’ negligence and attitudes were stated as reasons for non-adherence to ARV medications.

On lack of counselling, a patient stated

*“I defaulted because I was not educated about adherence. I did not know that I must take the treatment after I became pregnant.”*

The view of a 32-year-old woman explained how a health worker’s negligence influenced her adherence:

*“I defaulted because the nurse did not write down the TCA (to come again) date.”*

Then there was the case of two women whose anonymity was not respected: their neighbours worked at the clinics where they received their treatment and the neighbours talked about the patients’ HIV status to people thus illustrating how health workers’ attitudes and non-professional behaviour could impinge on adherence to medications.

Also, the response of a 32-year-old woman who stated that “*I lost my clinic book, and I went to the clinic, the nurse said I was lying about losing my clinic book and sent me home*” further illustrated how health workers’ attitudes can impinge on patients’ adherence to ART. This woman left and never returned until the index pregnancy.

## Discussion

This study brings together findings from a mixed methods study, which examined the levels and correlates of non-adherence and reasons for non-adherence among 1709 pregnant women infected with HIV in the Eastern Cape, South Africa. The analysis revealed that only 69.0% of HIV infected pregnant women self-reported complete adherence. Women who smoked during pregnancy were about two times more likely to report non-adherence than those who never smoked. Other risk factors for non-adherence were alcohol use during pregnancy, early booking, and non-disclosure of HIV status to sexual partner and a family member.

The non-adherence level among pregnant women in the Eastern Cape is high (31.0%) and warrants urgent interventions in view of the risk of mother-to-child transmission of HIV. The adherence level found in the current study is lower than those reported among rural pregnant women in Mpumalanga Province, South Africa [31], in Malawi [17], in Kenya [32], and in the USA [18]. However, lower adherence level was reported in Nigeria [33]. It is worth noting that our measure of adherence slightly differs from these studies. Nevertheless, the fundamentals of the adherence measures used in previous studies, which are self-reporting of non-adherence to ARV and defaulting in medication refill, are similar to this study. The previous studies compared were all among pregnant women on ART. The South African National ART guidelines recommended immediate education of patients on adherence to treatment at the time of diagnosis. Factors that influence non-adherence are dynamic and require different approaches as they change over time. The finding of this study is a scorecard of efforts to improve adherence among pregnant women infected with HIV in the Eastern Cape Province. Our findings clearly reveal that adherence level is sub-optimal for a significant proportion of the cohort and thus, indicates a need for urgent intervention.

Younger age, smoking, alcohol use, early booking, and disclosure to family and sexual partner were associated with ART adherence. Previous studies have reported the link between younger age and non-adherence [11, 12, 16]. However, younger age did not predict non-adherence in the logistic regression. The important independent predictors of non-adherence in our study setting were smoking, alcohol use, non-disclosure of HIV status to a family member, education and marital status. Smoking and alcohol use during pregnancy are unhealthy behaviours that are linked with poor pregnancy outcomes. Healthy behaviour such as, exercise and eating healthy, were shown to be linked with medication adherence [25, 34] though not reported in this study. There is, therefore, a need to give special attention to patients during antenatal care visits when they report unhealthy lifestyle behaviours, especially smoking and alcohol use. Medical staff should then promote healthy habits. A systematic review on barriers to ART adherence suggests that “forgetfulness” is the most reported reason for non-adherence [19], however, the underlying reasons why patients forget their medications were never investigated. The findings from the qualitative data (semi-structured interviews) reveal that partying and drinking were the main reasons patients forget to take their medications. Our finding is consistent with a Cape Town study that demonstrates that hazardous alcohol and drug use are associated with non-adherence and viral load non-suppression [35]. The results from our qualitative study help explain how alcohol use impedes on ARV adherence. Women often forget to use their medications when they go partying, especially on weekends.

Another important predictor of non-adherence is non-disclosure of HIV serostatus. The main reason for non-disclosure of HIV status is internalised stigma. The findings from the semi-structured interviews show how non-disclosure of HIV status impinges on medication adherence. Many HIV infected pregnant women stated that they could not use their medication without open disclosure of their HIV status. To avoid suspicion of their status, HIV infected pregnant women often travelled without their medications. Based on this finding, there is a need to identify patients who have not disclosed their status during antenatal care visits and counsel them, especially on the importance of adherence to medications. Previous studies have also reported non-disclosure as a barrier to ART non-adherence [15, 19, 21].

One surprising finding of this study is that women with grade 1–6 level of education were more likely to report complete adherence compared to women who had tertiary level of education. This finding is contrary to what was previously reported [36]. Based on the findings of the semi-structured interviews, the plausible explanation for this finding is busy work schedule, of which more educated women are most likely to be employed. One pathway through which work schedule could impact on medication adherence is that employed women may be unable to regularly refill their medications.

Another surprising finding of this study is that the proportion of women who were non-adherent was highest among women who booked early. The probable reasons for this finding could be due to pill fatigue [37] among those who booked early as a result of prolonged exposure to ARVs or simultaneous occurrence of early pregnancy symptoms such as nausea and vomiting. However, this was not established in this study.

An interesting finding of this study is that lack of food and money, treatments-related side effects, relocation, travel, forgetfulness, being busy at work and inadequate counselling are barriers to ARV adherence. The link between food insecurity and ART non-adherence has been reported and the lack of food worsens ARV side effects [38]. Participants’ narratives show that side effects are the most reported reason for non-adherence. This finding is consistent with previous studies [39–41]; however, a study shows that experiencing symptoms after starting treatment was not a barrier to adherence to ART [42]. The commonly reported side effect of ARV in this study was vomiting. Health providers should counsel pregnant women on what to do should they vomit following taking ARV. Also, pregnant women should be made aware that vomiting of ARV could have deleterious implications; decrease in drug concentrations and sub-optimal bioavailability with consequent risks of drug resistance. In addition, women should be educated about pregnancy symptoms (nausea and vomiting), which could occur concurrently following initiation of ART.

Another commonly reported reason for ART non-adherence is relocation and travel. This finding is consistent with a Malawian study [26]. HIV-infected pregnant women are turned down in other health facilities for lacking a transfer letter. Perhaps, providing medications pending the provisioning of transfer letter in all facilities in South Africa may improve adherence. Finally, the reasons many women were non-adherent are work-related. Workplace policy could be reviewed to favour HIV infected pregnant women, however, this is a distant solution. An immediate solution could be flexibility around the planning of medication refill dates and regular follow-ups of patients.

One important step towards improving ART adherence is identifying indicators of poor adherence [43]. Such indicators of poor ART adherence are useful resources for physicians to help identify patients who are in need of intervention to improve adherence. Based on the findings of this study, when HIV infected pregnant women report alcohol use, smoking, non-disclosure of serostatus and vomiting, physicians should have a heightened suspicion of the possibility of non-adherence to ART.

### Strength and limitations

The large sample size and the mixed methods study design ensured that the findings were reliable and representative of our study setting. However, the possible limitation of the study cannot be ignored. The study used self-reporting of non-adherence, which might have introduced social desirability bias. Though, there is no gold standard for measuring adherence in patients, the two item measures used in this study (even though demonstrated good internal validity), differs to many studies in the literature and thus may not allow for comparison.

## Conclusion

The high rate of non-adherence in a key population, such as HIV-infected pregnant women, is worrisome and portends a serious threat to the goal of elimination of mother-to-child transmission. Clinicians should proactively screen for lifestyle behaviours, HIV-associated stigma and ART side-effects at every antenatal clinic visit of pregnant women. Regular counselling on adherence and healthy lifestyle, and prompt management of ART side-effects would improve adherence in the study setting.

## Abbreviations

AOR: Adjusted odd ratio
ART: Antiretroviral therapy
ARV: Antiretroviral
CI: Confidence interval
HIV: Human immunodeficiency virus
PMTCT: Prevention of mother-to-child transmission
